# Paramedics’ perceptions of their scope of practice in caring for patients with non-medical emergency-related mental health and/or alcohol and other drug problems: A qualitative study

**DOI:** 10.1371/journal.pone.0208391

**Published:** 2018-12-13

**Authors:** Terence V. McCann, Michael Savic, Nyssa Ferguson, Emma Bosley, Karen Smith, Louise Roberts, Kate Emond, Dan I. Lubman

**Affiliations:** 1 Department of Nursing and Midwifery, Institute of Health and Sport, Victoria University, Melbourne, Victoria, Australia; 2 Turning Point, Eastern Health, Melbourne, Victoria, Australia; 3 Eastern Health Clinical School, Monash University, Melbourne, Victoria, Australia; 4 Queensland Ambulance Service, Brisbane, Queensland, Australia; 5 Ambulance Victoria, Melbourne, Victoria, Australia; 6 College of Medicine and Public Health, Flinders University, Adelaide, South Australia, Australia; 7 Department of Rural Nursing and Midwifery, College of Health, Science and Engineering, La Trobe University, Bendigo, Victoria, Australia; Yokohama City University, JAPAN

## Abstract

**Background:**

Paramedics are called on frequently to provide care to patients with mental health and/or and alcohol and other drug (AOD) problems, but may have mixed views about how this fits within their role.

**Aims:**

To explore paramedics’ experience of caring for patients with non-medical emergency-related mental health and/or AOD problems, understand their perceptions of their scope of practice in caring for these patients, and ascertain if their practice should be extended to incorporate education with these patients.

**Method:**

A convenience sample of 73 paramedics from most Australian states and territories—recruited through an online survey—participated in individual audio-recorded, qualitative interviews, conducted by telephone. The interviews were part of a mixed method study comprising qualitative interviews and online survey. A Framework Method of analysis to analyse the qualitative data.

**Results:**

Three themes and sub-themes were abstracted from the data about participants’ experiences and, at times, opposing viewpoints about caring for patients with non-medical emergency-related mental health and/or AOD problems: caring for these patients is a routine part of paramedics’ work, contrasting perspectives about scope of practice in caring for this group of patients, competing perspectives about extending scope of practice to incorporate education with this cohort of patients.

**Conclusions:**

Paramedics need more undergraduate and in-service education about the care of patients with mental health and/or AOD problems, and to address concerns about extending their scope of practice to include education with these patients. Thought should be given to introducing alternative models of paramedic practice, such as community paramedicine, with a focus on supporting people in the community with mental health and/or AOD problems. There is a need for a change in workplace and organisational culture about scope of practice in caring for patients with these problems. Extending paramedics’ role could, potentially, benefit people with these problems by improving the quality of care, reducing the need for transportation to emergency departments, and decreasing clinicians’ workloads in these departments.

## Introduction

Paramedics frequently care for patients who have mental health and/or alcohol and other drug (AOD) problems, which represent a substantial part of their workload [1–3]. For instance, in the state of New South Wales in 2013, NSW Ambulance attended more than 60,000 calls classified at the call-taking stage as mental health or psychiatric [4]. However, there is limited research on how paramedics feel about providing care to patients with non-medical emergency-related mental health and/or AOD problems, and what they believe their role is or should be in this context [5]. This is an important consideration because perceptions of what practices are within, or beyond, paramedics’ scope of practice are likely to influence the nature and extent of the care provided.

The relatively small body of literature on paramedics’ perception of their role in relation to patients with non-medical emergency-related mental health and/or AOD problems provide some tentative clues. Roberts and Henderson [6] conducted a mixed method study (survey (n = 74) & 3 focus groups of paramedics; ambulance service clinical database) with South Australian Ambulance to examine workforce perspectives about attending patients with known or suspected mental illness. The results indicated that paramedics viewed their role as primarily one of transportation in this situation, they felt ill-prepared to manage and care for these patients, and needed tailored education. The authors argued that de-institutionalisation of mental health care and under-investment in community services in places like Australia for people with mental health and/or AOD problems has positioned paramedics as frontline community care providers.

A qualitative study (observations and interviews) by Prener and Lincoln [2] in the US found that urban paramedics and emergency medical technicians were frustrated by what they perceived as “filling the gaps for other health care and social services” in attending to patients with mental health and/or AOD problems. Similarly, studies have documented a perception amongst paramedics that non-medical emergency-related mental health and/or AOD presentations divert resources and attention away from the ‘real’ medical emergency work that paramedics believe should be the primary focus of their work [2, 6].

A range of initiatives have been undertaken to build the capacity of paramedics to perform extended and advanced care roles, including providing preventative care [7] and more holistic care [8]. While these efforts have had some impact on paramedics’ roles [7], they do not appear to have extended to mental health and/or AOD care. For instance, like Prener and Lincoln [2], Roberts and Henderson’s [6] study of paramedics in South Australia found that paramedics overwhelmingly (n = 73, 98.6%) reported transportation of patients to emergency care as a key part of their role when faced with mental health presentations. In part, this may be influenced by clinical practice guidelines and legislative responsibilities that sanction/encourage involuntary mental health care (and thus transportation) in instances where patients are deemed to pose an immediate threat to themselves or others [5, 9], and a lack of availability of alternative referral options for patients with mental health and/or AOD problems. However, other potential functions, such as communicating with the patient, mediation, providing treatment, or referral to services are reported less frequently [6].

Rees, Rapport and Snooks [10] conducted a constructivist meta-synthesis of 12 qualitative studies of paramedics and emergency department staffs’ perceptions about caring for patients who self-harmed. They reported that although none of the studies focused specifically on self-harm, paramedics perceived their primary role was to provide medical care and preserve life, and patients requiring medical care were considered a higher priority than those who self-harm. They also had difficulty and anxiety about the application of mental health acts in their practice, and there was a dichotomy between policies and guidelines advocating a potential for a paramedic role with patients who self-harm and paramedics’ awareness of such potential.

Several other contextual factors beyond paramedics’ individual attitudes have also been identified as potentially shaping their work practices in caring for patients with mental health and/or AOD problems. For instance, high work pressures and limited mental health training may constrain the ability of paramedics to respond to the needs of patients with these problems beyond transportation [2, 5, 9, 11–14]. Alternatively, organisational cultures in ambulance services that promote a medical emergency care focus can, potentially, reinforce the idea of responding to non-medical emergency-related mental health and/or AOD presentations as being a less important aspect of paramedics’ role [2, 6]. Furthermore, such ideas may be reinforced and/or reflect the broader stigmatisation of people with these problems [2, 15, 16].

While the small body of literature on paramedics’ perception of their role in caring for patients with non-medical emergency-related mental health and/or AOD problems provide some useful insights, there are several gaps that are worth noting. First, most empirical studies have focused on particular locations or states within a country, which limits the applicability of their findings beyond those settings. Second, few have been sensitive to differences in perceptions or experiences of paramedic role between different groups of paramedics, such as those from rural or metropolitan areas. Third, few have documented perceptions around whether paramedics feel that their scope of practice should be extended. In light of these gaps, and the need for further research on the role of paramedics in providing mental health care [5], the aims of this paper are to explore the experiences of paramedics across Australia in caring for patients with non-medical emergency-related mental health and/or AOD problems, understand their perceptions of scope of practice in caring for these patients, and ascertain if their practice should be extended to incorporate education with this cohort of patients.

## Methods

The qualitative findings presented in this paper were part of a larger mixed method study involving an online survey and qualitative interviews with paramedics across Australia. The online survey examined paramedics’ attitudes toward men who presented to ambulance services with mental health and/or substance use problems; and assessed the barriers, facilitators and professional development needs of paramedics in providing support to men with these problems. A separate part of the study involved qualitative interviews with men who had presented to these services with these issues.

### Participant recruitment

Initially, all paramedic services throughout Australia were approached seeking permission to conduct the study. Of these, six (of the eight) states and territories (New South Wales, Northern Territory, Queensland, South Australia, Tasmania and Victoria) gave permission to invite paramedics employed by their services to participate in the study. Recruitment occurred across metropolitan, regional and rural sites in these services. Several approaches were adopted to recruit paramedics, including emails, e-bulletins and e-newsletters sent by their ambulance service, Paramedics Australasia’s (the peak professional organisation representing paramedics in Australia) newsletter and website, and paramedic social media. Interested paramedics were asked to complete an online survey. After completing the survey (n = 1,230), a final question asked if they would be willing to take part in a qualitative interview. If interested, they were requested to provide telephone contact details, which were stored in a separate database to their survey responses. A follow-up telephone call was then made to each prospective participant to explain the qualitative part of the study and answer their questions. A copy of the participant information and consent form was emailed. If still interested in participating, arrangements were made to conduct the interview with a convenience sample of 73 paramedics. Five human research ethics committees in three states and territories gave ethical approval to undertake the study (Eastern Health Human Research Ethics Committee (Victoria), Monash University Human Research Ethics Committee (Victoria), South Eastern Sydney Local Health District Human Research Ethics Committee (New South Wales), South Australia Department for Health and Wellbeing Human Research Ethics Committee, Flinders University Social and Behavioural Research Ethics Committee (South Australia)), while three other states/territories accepted ethics approval from another state. Informed consent to participate was obtained prior to the commencement of the interviews, and was audio-recorded via telephone, as approved by the ethics committees.

### Data collection

Individual interviews were conducted, using a semi-structured interview schedule. The interview questions focused, generally, on paramedics’ experience in dealing with patients with mental health and/or substance use problems, differences in the way they dealt with patients with these issues in comparison to those with medical issues, perceived role and previous training in dealing with patients with mental health and/or substance use problems, and their views about extending their scope of practice in dealing with patients with these issues. Interviews were audio-recorded and undertaken by telephone, and, apart from the interviewer and participant, no-one else was present. To enhance rigour, interviewers completed interviewer training, were asked to adhere flexibly to the interview guide, and, along with the researchers, underwent a review process of the initial audio-recorded interviews. Each interview lasted an average of 56 minutes (range 27 to 79 minutes). Because of the data collection method, transcripts were not returned to participants for comment. The interviewers had no previous contact with participants. Two graduate female researchers, NF (Research Assistant) and KE (Lecturer), who were given prior training by MS, conducted most interviews. MS, Ph.D. (male, Research Fellow), conducted a small number of interviews. The interviewers were aware of literature indicating that paramedics had mixed beliefs about caring for people with mental health and/or AOD problems, but were open-minded about the reasons paramedics had for these contrasting views.

### Data analysis

Interviews were analysed in NVivo (Version 11) [17] in accordance with the Framework Method [18, 19]. The Method is particularly useful when analysing data in qualitative studies with large numbers of participants, as in the present study. Apart from enabling themes to be abstracted inductively, the Method commences deductively from the pre-determined aims of the research, which makes it a particularly suitable approach for applied or policy relevant research [20]. Seven steps were involved in this process: (i) verbatim transcription, reading and re-reading interview transcripts; (ii) initial coding of several transcripts; (iii) development of an analytical framework for coding based on initial coding of several transcripts; (iv) applying the analytical framework to subsequent transcripts, with emergent themes from the interview data being added to the framework during this process; (v) charting data into the framework matrix; (vi) summarising respondents’ responses across each theme; and (vii) interpreting final themes in light of what they intimate about paramedic’s scope of practice [20].

Theme saturation with ‘thick’ description of the data was accomplished when no new data were identified to add to these themes [21]. Theme saturation, an important issue in the rigour of qualitative studies, also determined the sample size of the study [22]. Use of exemplars in this paper to illustrate each theme aids transferability of the findings by enabling readers to appraise the data collection process and findings and their transferability to other paramedic workplace settings [21]. Other approaches used to enhance rigour included analyst triangulation, whereby all authors reviewed findings; consideration of negative cases; inclusion of multiple perspectives; and use of an audit trail for reflexivity [23]. Initial analysis was undertaken by NF, KE and MS, followed by an independent review of the process by TMcC, which strengthened the rigour of the study [24]. Differences in coding and theme identification were resolved through discussion until consensus was achieved. Semantic analysis progressed from summary and description in the results section, to interpretation of the results in the discussion section [25].

## Results

### Participant characteristics

In total, 73 paramedics, with a mean age of 43.9 years, participated (Table 1). Almost two-thirds were male. Participants worked in 6 of the 8 Australian states and territories. Just over 40% worked in metropolitan cities, while the remainder worked in regional and rural centres, and, to a lesser extent, remote areas.

**Table 1:** Paramedic participants’ sociodemographic and workplace details (N = 73).

	n	%
Gender		
Male	42	64.6
Female	23	35.4
**State/Territory of work**		
New South Wales	22	30.1
Northern Territory	8	11.0
Queensland	11	15.1
South Australia	5	6.8
Victoria	19	26.0
Tasmania	8	11.0
**Geographic location of work**		
Metropolitan	32	43.8
Regional	17	23.3
Rural	20	27.4
Remote	4	5.5
**Cultural background/Place of birth**		
Aboriginal & Torres Strait Islander (ATSI)	2	2.8
Born in Australia but non-ATSI	53	72.6
Born outside Australia	18	24.6
**Highest professional educational qualification**		
Non-university vocational training certificate	1	1.4
Diploma	20	27.4
Degree	32	43.8
Graduate Certificate	6	8.2
Graduate Diploma	13	17.8
Master’s Degree	1	1.4
	Mean	Range
**Age** (years)	43.9	23–63

### Themes

Three interrelated themes were abstracted from the data, reflecting participants’ experiences and views about caring for patients with non-medical emergency-related mental health and/or AOD problems: (i) caring for these patients is a routine part of paramedics’ work, (ii) contrasting perspectives about scope of practice in caring for this group of patients, (iii) competing perspectives about extending scope of practice to incorporate education for this cohort of patients.

### Caring for patients with non-medical emergency-related mental health and/or AOD problems is a routine part of paramedics’ work

This theme highlights that caring for patients with non-medical emergency-related mental health and/or AOD problems has become an increasingly routine part of paramedics’ work in recent decades. The nature of this work contrasted with their routine work historically, where it was less common to encounter individuals with these problems. It was also evident that paramedics were providing care to patients with a wide range of mental health and/or AOD problems, from non-medical emergency-related mental health and/or AOD specific problems, to medical problems secondary to mental health and/or AOD problems. They were caring for patients across the acute-to-chronic spectrums of mental health and/or AOD problems, and across all geographic settings, from metropolitan to rural and remote locations.

*Across the board we see mental health problems*, *personality disorders and then definitely drug and alcohol associated problems*. *We would see that nearly every day that we're working*, *at least one person a shift*, *and we've got a couple of crews running at [regional town]*, *so everybody would have one of those at least every shift*, *if not more*. (Interview 23, female, regional workplace, aged 49 years)

In commenting about their increasing experience of caring in this context, participants attributed this primarily to a shortage of community and inpatient services for patients with these problems. A direct consequence of this shortage was an increase in demand for paramedic services and presentations at emergency departments. While there were several initiatives to bridge the gap in the provision of medical resources between rural and metropolitan areas, this did not extend to providing adequate community and inpatient services to meet the needs of people with mental health and/or AOD problems. Hence, a shortage of appropriate services was one of the reasons cited for transportation of this group of individuals to emergency departments.

*We're introducing all sorts of things to try and even out their health discrepancies between rural and metropolitan areas*, *like for acute heart attacks and rapid transport to a* [cardiac] *Cath* [catheterisation] *lab*, *trauma systems so that people get to a trauma unit as quickly as possible with a helicopter*. *But we don't seem to have the same level of resourcing for people with acute psychiatric conditions and they're missing out …*. *You don't want to be a public psychiatric patient in the country; it's just horrible*. (Interview 55, female, rural workplace, aged 59 years)

There was also acknowledgment that current paramedic services were ill-equipped to meet the needs of, and respond appropriately to, people with non-medical emergency-related mental health and/or AOD problems within their existing medically-focused workloads. One way of responding to the increasing demand was the suggestion to establish a paramedic sub-service specifically to provide care for people with these problems.

*A while back*, *I actually sent an email to my team manager saying because the high level of mental health cases we do*, *we should have a mental health ‘truck*,*’ like a mental health ambulance solely for dealing with mental health [patients]*. *I would go and work on that*, *no trouble*. *But … the government wouldn’t budget for that anyway*. (Interview 56, female, rural workplace, aged 51 years)

### Contrasting perspectives about scope of practice in caring for patients with non-medical emergency-related mental health and/or AOD problems

In this theme, it was evident that participants had two competing perspectives about whether providing care to patients with non-medical emergency-related mental health and/or AOD problems was within their scope of practice. These perspectives were reflected in two contrasting sub-themes about their practice in this context: caring for patients with non-medical emergency-related mental health and/or AOD problems is an integral part of practice, and responding to patients with non-medical emergency-related mental health and/or AOD problems is outside scope of practice.

#### Caring for patients with non-medical emergency-related mental health and/or AOD problems is an integral part of practice

In this sub-theme, participants emphasised that caring for patients with non-medical emergency-related mental health and/or AOD problems was an integral part of their practice, and individuals with these problems were equally deserving of care as those with acute medical problems. In this view, no distinction was made in the quality of care provided to those with non-medical emergency-related mental health and/or AOD problems and those whose primary concerns were medical problems. While the nature of the care provided differed, the care and commitment to care was the same.

*I feel the same way in that people with genuine mental health problems are just as deserving of medical help from me or the* [paramedic] *system as anybody with any diagnosed or any other medical problem*. *No more*, *you know; just the same as treating someone with a broken leg or having a myocardial infarction*, *and treating them and seeing a result*. (Interview 25, male, regional workplace, aged 44 years)

Family and personal experience as well as educational and clinical role models helped influence participants’ views about caring in this context being within their scope of practice.

*I'm fairly comfortable and confident dealing with the different situations*. *Bit of personal history I suppose and insight into some of the conditions*, *also had family members with mental conditions…*. *When I did mental health at university* [in the paramedic course], *my lecturer was very passionate about it and very knowledgeable and I had some lengthy conversations with her about different treatment options and the way to talk to people*. (Interview 10, male, regional workplace, aged 37 years)

*If you were lucky*, *you were put with an experienced officer and I had been fortunate*. *I'd been working with officers that had been quite a few years in the job and they taught me a lot*. (Interview 24, male, regional workplace, aged 52 years)

Overall, despite the limited time they had to engage in mental health-related work, this sub-theme highlights the emphasis that some paramedics placed on addressing mental health and/or AOD problems.

#### Responding to patients with non-medical emergency-related mental health and/or AOD problems is outside scope of practice

In this sub-theme, participants indicated that caring for patients with non-medical emergency-related mental health and/or AOD problems was beyond their existing scope of practice. Three influences contributed to this perception: (i) medically-oriented role, (ii) time constraints precluding engagement, and (iii) lack of education about caring in this context.

Medically-oriented role There was a distinct perception among many paramedics that caring for patients with non-medical emergency-related mental health and/or AOD problems was not within paramedics’ scope of practice. Patients with these problems were perceived as more difficult to deal with and less deserving of paramedic care than patients with acute medical problems. There was also a lack of understanding of the seriousness of mental health and/or AOD problems.

*I find them really challenging*, *purely from the perspective that I am not qualified to make a formal decision or diagnosis that you're faking it and you're full of ‘crap*.*’ I find that challenging because of the taking resources away from somebody else that might need it*. (Interview 22, male, metropolitan workplace, aged 40 years)

Patients with mental health and/or AOD problems were also perceived as only deserving of paramedic care in the event of medical, possibly life-threatening, emergencies occurring as a result of their mental health and/or AOD problems. However, this still suggests a reluctance to deal with these patients with these problems.

*I think paramedics should only be involved where an episode of harm has occurred*, *because there is something that needs to be done for them in a physical sense and also in an emotionally supported sense*. *Where it is just because somebody needs somebody to talk to*, *I don't think that is our role*. *That is what mental health services are for*. (Interview 1, male, metropolitan workplace, aged 45 years)

What these exemplars highlight, overall, is that participants perceived their scope of practice as being focused primarily on providing medically-oriented emergency care.

Time constraints precluding engagement There was a common perception that time constraints precluded more in-depth engagement with patients with non-medical emergency-related mental health and/or AOD problems. These constraints were perceived as being attributable to paramedic service pressure to be available to respond to other waiting cases and limited time allocated to individual call-outs. There was acknowledgment by some participants, however, that it was still possible for paramedics to make a helpful contribution in the limited time that had available to provide care in this circumstance.

*… we don't get the time these days*. *They're* [paramedic managers] *always pushing us as a service that we need to be hitting these targets and we need to be getting out* [back into the] *community and things like that*. *But I think slowing down and taking time is a huge impact on the way that people will then behave with us*. (Interview 73, female, metropolitan workplace, aged 39 years)

*Occasionally*, *you might attend to a patient who is depressed and potentially suicidal and voluntarily going to hospital*, *so that there's more opportunity then* [to provide advice]. *But then*, *even still*, *there's limited time*, *especially working in the metropolitan region*, *the transport's short and it's only really during transport that you've got that opportunity to have a chat to them …* (Interview 36, male, metropolitan location, aged 28 years)

Lack of education about mental health and AOD problems A common comment was that lack of pre-service employment education (now university-based undergraduate) and in-service education about caring for patients with non-medical emergency-related mental health and/or AOD problems contributed to a workplace culture and discourse that emphasised adopting an emergency medical care-oriented scope of practice. The limited amount of time devoted to mental health and AOD problems in pre-service employment curricula was disproportional to the frequency of encounters with patients with these problems in paramedics’ day-to-day practice. When pre-service employment education was provided, it was delivered sometimes by lecturers with little or no expertise in mental health and AOD problems. Hence, participants indicated that they had inadequate background knowledge and skills to respond appropriately in this situation, and, generally, this deficit was reinforced by a lack of in-service education to address this shortfall. However, some commented that they had received a limited amount of in-service mental health education from mental health nurses, but considered this insufficient to equip them carry out their routine work with patients with such needs.

*So*, *we had very*, *very little* [undergraduate] *mental health training*, *and then we go out into the wide world*. *You know*, *you're seeing in some areas*, *at least 10 per cent*, *10*, *15 per cent of your jobs are mental health-related*, *or 20 …* [with] *drug and alcohol things …*. *Just about all the* [in-service] *training we've had since is basically because they recognised it* [undergraduate training] *was so poor*, *I guess*, *and has been* [provided] *within the service*. (Interview 4, male, rural workplace, aged 40 years)

### Competing perspectives about extending scope of practice to incorporate education

In this theme, participants had contrasting perspectives about whether their scope of practice should be extended to encompass provision of education to people with non-medical emergency-related mental health and/or AOD problems being transported to an emergency department, metal health or AOD service and to those not being transported. Not being transported in this context refers to paramedics making a clinical decision to manage patients in their homes instead of transporting them to an appropriate service. The contrasting perspectives were manifested in two competing sub-themes about the nature of their practice in this context: extending practice to incorporate education, and maintaining existing emergency medical care-focused scope of practice.

#### Extending practice to incorporate education

In this sub-theme, participants highlighted that there was scope for paramedics to extend their existing medical-oriented role to encompass education to patients with non-medical emergency-related mental health and/or AOD problems being transported and to those not being transported. Participants claimed that this would add to their existing educational role with patients with non-acute medical conditions who were not being transported. Extending their role in a similar way with patients with non-medical emergency-related mental health and/or AOD problems could, potentially, lead to a reduction in paramedics’ current workload in transporting patients with these problems to emergency departments and mental health services and, indirectly, to the workloads of emergency department clinicians.

*I think the potential for us to provide information is huge*, *because we do a lot of referral and management advice as part of our normal role with patients with medical issues*, *chronic illnesses or non-acute medical issues*. *We have access to phone numbers and services that we can help these people with*. *With mental health*, *we have nothing …*. *we don't have access to anything*. (Interview 57, male, metropolitan workplace, aged 25 years)

Education was also conceived as being beneficial to patients with these problems, but explicit in this sub-theme was concern about a lack of alternative mental health and AOD services and acknowledgment of the limitations of their current transportation role with individuals with these problems.

*We never go past accident and emergency … we cannot ring the mental health ward directly and have them coming in directly*. *It has to go through accident and emergency*. *A lot of people get sent back on their way again*. *There's an enormous amount of people that never make it to the mental health ward simply because there's no place* [for them]. *People who you think should stay or should take their drugs*, *they go home*. (Interview 11, female, metropolitan workplace, aged 52 years)

In expressing a commitment to adopt an educative role, there was also recognition that paramedics were not well-equipped to provide the type of information and support required in this situation to affected individuals and their families. In their current practice, such information was provided mainly on unplanned basis by individual paramedics rather than a service-wide and systems approach being adopted to address this issue.

*I have given people advice in terms of ringing BeyondBlue*, *for example*, *… give them the phone number to call that they can talk to people*. *I know there're probably other services available that we could use for drug and alcohol*, *but I haven't referred any people on to those before*. *Again I think it's a case of making that information available to paramedics and saying*, *“look*, *these services are available*. *Hand on the information*.*”* (Interview 10, male, regional workplace, aged 37 years)

#### Maintaining existing emergency medical care-focused scope of practice

In this sub-theme, participants emphasised that their scope of practice should not be extended to incorporate education to patients with non-medical emergency-related mental health and/or AOD problems being transported and to those not being transported. Instead, paramedics should maintain their existing emergency medical care-focused role, whereby they transported patients with non-medical emergency-related mental health and/or AOD problems to emergency departments and, in some circumstances, mental health services. In acknowledging that the emergency medical care-oriented part of their role had diminished over time, participants indicated, however, that they did not conceive that the non-emergency part of their role should also incorporate education with patients with these problems. Instead, they believed that the educative role should continue to be the responsibility of accessible mental health services.

*As far as giving the person advice on how to manage their condition*, *that's not really our role …*. *There should be somewhere in the community where this advice is already available and that person should be able to seek that without having to call emergency* [paramedic] *services*. (Interview 66, male, metropolitan workplace, aged 50 years)

It was also evident that organisational, legal and safety constraints in certain jurisdictions influenced some participants’ preference to maintain their current emergency medical care-focused transportation role. Operating beyond this proscribed role in these circumstances was clearly not a consideration.

*Our …*. *(name of paramedic service) has a big fear of the Coroner*, *and that’s because of things that have happened in the past that haven’t really been the paramedics fault*. *But the service has been so neglectful in those situations*, *I suppose that they’ve had a massive knee-jerk reaction …*. *Everyone goes to hospital*. *If you call us*, *you’re going to hospital*, *no matter what*. *It’s just not worth losing my job over people* [with mental health and/or AOD problems] *who don’t care about themselves*. (Interview 12, male, rural workplace, aged 36 years)

These exemplars illustrate, overall, that this group of participants conceived their scope of practice, when dealing with patients with non-medical emergency-related mental health and/or AOD problems, as predominantly about transporting them safely to an emergency department, attending to any related emergency medical problems, but not about educating and engaging with them in any overtly therapeutic way to address their mental health and/or AOD problems.

## Discussion

The aims of this paper were to examine paramedics’ experience of caring for patients with non-medical emergency-related mental health and AOD problems, ascertain their beliefs about scope of practice in caring for these patients, and establish if they believed that their practice should be extended to include an educative role with these patients. Three interconnected themes were abstracted from the data illustrating participants’ experiences and, at times, contrasting views about caring for patients with these problems.

The first theme highlighted that, in contrast to historical experiences, caring for patients with non-medical emergency-related mental health and/or AOD problems is now a routine part of paramedics’ work. This finding is consistent with those of other studies reporting that paramedics frequently care for patients with mental health and/or AOD problems and transport them to emergency departments [1–3]. Possible explanations for this situation are that the de-institutionalisation of mental health care and under-investment in community services in developed countries, such as Australia, for people with mental health and/or AOD problems have made it increasingly difficult for these individuals to access specialised care and treatment [6]. Another explanation for the increase in AOD presentations is liberalisation of the alcohol market [26], and drugs that cause greater harm are more likely to lead to an emergency call for ambulance services (e.g., methamphetamine, synthetic drugs, overdoses related to prescription opioids) [1]. A consequence of this situation is that many people with mental health and/or AOD problems turn to paramedic services for help and to transport them to emergency departments, which is an indirect way of accessing specialised services.

Another consequence of this situation is that it has led to an extension of paramedics’ routine work to include caring for and transporting patients with mental health and/or AOD problems [3]. In the present study, there was also recognition of the limitations of the current generic model of practice, whereby paramedics responded to call-outs to people with a broad spectrum of medical, mental health and/or AODs problems. There were calls for the introduction of alternative models of practice, including a mental health and/or AOD specific model, which may have particular merit in jurisdictions experiencing high demand for this type of service, such as in metropolitan centres. A proof of concept of this type of model was undertaken in Sydney, incorporating an outreach team comprising a paramedic and a mental health nurse [27]. Initial findings indicated that almost 70% of patients with mental health and/or AOD problems were not transported to emergency departments, just over 45% were brought directly to a mental health facility, and almost 22% were not transported. A variant of this type of model is the use of mental health clinicians working in call-taking and dispatch. Limitations of this type of model employing mental health clinicians, however, are it may result in under-utilisation of these personnel and reinforce a perception among some paramedics that dealing with patients with non-medical emergency-related mental health and/or AOD problems is not within their scope of practice.

The second theme indicated that there were contrasting perspectives about scope of practice in caring for patients with non-medical emergency-related mental health and/or AOD problems. Some paramedics perceived their scope of practice as incorporating the care of patients with these problems in their day-to-day practice, and these individuals were as deserving of care as those with medical conditions. Family and personal experience, and the influence of training experiences with professional role models (educational and clinical), contributed to this broader perception of their scope of practice. In contrast, other participants considered that caring for individuals with these problems was beyond their scope of practice, and that these individuals were difficult to deal with and less deserving of paramedic care than patients with acute medical problems. One explanation for this finding is public stigma towards people with mental health and/or AOD problems contributed to paramedics’ reluctance to broaden their scope of practice in this context. Paramedics reside in the broader community in which they work, and their attitudes are reinforced by, and reflective of, such communities, where public stigmatisation of people with people with mental health and/or AOD problems occurs routinely [2, 15, 16].

Another explanation for this finding is that inadequate undergraduate and in-service mental health education and training constrained paramedics’ confidence, ability and willingness to engage therapeutically with patients with mental health and/or AOD problems [2, 5, 9, 11–14]. A third explanation is high workloads and pressure from paramedic services to be available to respond to waiting cases precluded a desire to broaden their existing scope of practice.

The third theme highlighted that there were competing perspectives about whether paramedics’ scope of practice should be extended to incorporate education to patients with non-medical emergency-related mental health and/or AOD problems, and that there should be increased options for alternative treatment pathways for these patients. One group of participants emphasised that their practice should be extended to encompass education in this circumstance. Extending their scope of practice could be interpreted as an acknowledgment, in this and other studies [1–3], that an increasing proportion of paramedics’ day-to-day practice involves caring for patients with these problems. Therefore, extending their practice in this context could be interpreted as a natural extension of paramedics’ existing educative role with patients with medical problems who are not transported to hospital. In light of the level of unmet needs of people with non-medical emergency-related mental health and/or AOD problems in the community, there may also be scope to introduce and evaluate rigorously a second alternative model of practice, this time incorporating an adapted form of existing community paramedicine practice.

Community paramedicine, originally introduced in the US, but also operating now in other countries, such as Canada, the UK and Australia, is where paramedics (mostly) are trained to provide assessments and interventions in the community but normally do not provide acute transport [28, 29]. Generally, community paramedicine programs focus on social support, wellness, prevention, care of people with chronic illness and increasing medical adherence [28]. Within the context of the present study, there is scope to broaden community paramedicine roles to include people with non-medical emergency-related mental health and/or AOD problems who do not require transportation to emergency departments, mental health or AOD services. Instead, they would be managed in their homes, followed up and/or referred to, for example, a GP, mental health or AOD service.

Another group of participants in the present study indicated that their existing emergency medical care-oriented scope of practice should be maintained and not extended into an educational role with patients with non-medical emergency-related mental health and/or AOD problems. This finding is consistent with those of other studies, where paramedics claim that their primary role with patients with these problems is to transport them safely to hospital emergency care departments [2, 6]. Lack of access to, and availability of, specialised care and treatment services for people with non-medical emergency-related mental health and/or AOD problems, means that paramedics have to meet this shortfall, and is one explanation for their preference to maintain their existing medical-oriented role.

Roggenkamp et al. [3], in an analysis of mental health-related presentations to emergency medical services in the state of Victoria, Australia, reported that one-in-ten presentations to these services were mental health-related and, of these, most were transported without any treatment from paramedics. The authors highlighted the need to provide alternative models of care which improved access to specialised mental health services for these patients. Lack of service access, along with stigma, can reinforce a perception that having to deal with patients with these problems diverts resources and attention from, what paramedics believe should be the main focus of their work, caring for patients with acute medical problems [2, 30]. Furthermore, and in line with the findings of the present study, paramedic service policies and clinical practice guidelines, which are informed by legislation that enables involuntary mental health care and transportation of patients considered an immediate threat to themselves or others [5, 9], may also reinforce paramedics’ perception that their role in this situation is primarily to provide transportation.

Other explanations for maintaining their current emergency medical-oriented scope of practice are, overall, a lack of undergraduate and in-service education and training about the care of patients with mental health and/or AOD problems, and ambulance organisational cultures and discourses that reinforce a medically-oriented primary focus [2, 6, 30]. Perpetuating this focus could also be interpreted as a means of enhancing the professionalisation of paramedic services, something that is less likely to be achieved by broadening their scope of practice to include an educative and non-transportation role with patients with mental health and AOD problems. Paradoxically, broadening their scope of practice to be able to independently manage patients with non-medical emergency-related mental health and/or AOD problems in their homes (instead of primarily being a transportation service), could be conceived as a way of increasing the professionalisation of paramedic services.

### Limitations and strengths

The findings of this large qualitative study are context bound to the participants and settings in which it was undertaken [31]: paramedics practising in metropolitan, regional and rural settings in most Australian states and territories. Even though generalisability is not prerequisite for conducting qualitative research,[32] the themes can be verified [33–35] and, as our discussion highlights, are applicable to Australian paramedics who did not participate in the study and those in other developed countries. A potential limitation of the study is the grouping of mental health and AOD problems in questions limited the ability to tease one from the other. Nevertheless, there were no discernible differences evident in the data regarding participants’ perspectives about their scope of practice relating to patients with these problems. Another limitation is that there are some variations in scope of practice, policies and workplace cultures across Australian ambulance services.

### Implications

Our findings suggest that paramedics’ views about their scope of practice in caring for patients with mental health and/or AOD problems are reflective of educational and workplace cultures, organisational policies and concerns, and public stigma towards people with these problems. Hence, a broad range of measures are needed to address these influences. From an educational perspective, more emphasis needs to be placed in paramedic undergraduate curricula and in-service education programs to better equip paramedics to care for patients with these problems. From a paramedic service workplace organisational and cultural perspective, consideration should be given to reviewing policies and clinical practice guidelines to ensure they provide a framework that reflects a broader view of practice, beyond the current medically-focused scope of practice. Given the scale of the issues that ambulance services face with these patients, consideration should also be given to introducing alternative models of practice that incorporate skilled mental health clinicians working alongside paramedics as well as in call-taking and dispatch. Similarly, consideration should be given to introducing community paramedicine with a focus on educating and supporting people in the community with non-medical emergency-related mental health and/or AOD problems. Simultaneously, it is important to promote a workplace discourse that encourages and supports paramedics to adopt a broader range of practice that incorporates a holistic approach to caring for patients with mental and/or AOD problems. Extending paramedics’ scope of practice in these circumstances could, potentially, benefit people with mental health and/or AOD problems and lead to a reduction in the number of people requiring transportation to emergency departments as well as a reduction in the workloads of clinical staff in these departments. From a mental health and AOD service perspective, more attention needs to be giving to increasing access to, and provision of, these services in regional and rural areas in particular. Finally, future research needs to evaluate if these recommendations, if adopted, result in better outcomes for people with mental health and/or AOD problems.

## Supporting information

S1 Table Consolidated criteria for reporting qualitative studies (COREQ): 32-item checklist. (DOCX)
